# Case Report: A Case Series Linked to Vitamin D Excess in Pet Food: Cholecalciferol (Vitamin D3) Toxicity Observed in Five Cats

**DOI:** 10.3389/fvets.2021.707741

**Published:** 2021-08-18

**Authors:** Carla G. Vecchiato, Costanza Delsante, Giorgia Galiazzo, Simone Perfetti, Carlo Pinna, Maria C. Sabetti, Laura Zagnoli, Giacomo Biagi, Marco Pietra

**Affiliations:** ^1^Department of Veterinary Medical Sciences, University of Bologna, Ozzano dell'Emilia, Italy; ^2^Clinica Veterinaria dell' Orologio - AniCura, Sasso Marconi, Italy

**Keywords:** hypercalcemia, cholecalciferol intoxication, feline nutrition, cat food, case report, vitamin D3

## Abstract

Cholecalciferol (vitamin D3) toxicity caused by defective pet food formulations is a rare occurrence described in cats. Nevertheless, it poses a health risk, even though the affected pet food is not fed as the sole diet. Excessive vitamin D3 intake might cause hypercalcemia and soft tissue mineralization, which are findings that prompt clinicians to further investigate the feasible etiology. This case series describes the effects of an extremely high vitamin D3 intake in five young cats caused by the consumption of a fish-based complementary kitten pet food (KPF) that was fed to all of the cats as part of their diet (cases 1, 2, and 3) or eaten exclusively (cases 4 and 5). Due to the different amounts of vitamin D3 consumed, diagnostic examinations showed different degrees of severity of hypercalcemia and azotemia as well as different radiographic findings in cases where diagnostic imaging was performed (cases 2, 4, and 5). All of the cats were treated by withdrawing the affected food and providing medical management of the hypercalcemia. All of the cats recovered, except for two persistent azotemic cats, which developed chronic kidney disease. The goal of this case series is, therefore, to describe the occurrence and resolution of an acute vitamin D3 toxicity due to the highest amount of dietary vitamin D3 intake that has ever been described in domestic cats.

## Introduction

Vitamin D is present in two main forms: vitamin D3 and vitamin D2. The latter is found in small amounts in a plant-based diet because vitamin D2 is produced by fungal and yeast contamination on plants by the UVB exposure of ergosterol, while vitamin D3 can be obtained from animal products since it originates in skin cells by exposure to sunlight 7-dehydrocholesterol (1–3). Domestic cats can utilize vitamin D2, but to a lesser extent than vitamin D3, due to a lower affinity of vitamin D binding proteins to vitamin D2 metabolites compared to vitamin D3 (4). Nevertheless, as is the case for most terrestrial carnivores, they do not synthesize vitamin D3 by way of the skin and, therefore, they are dependent on the dietary intake of vitamin D (5, 6). Vitamin D3 is rapidly absorbed after ingestion and is firstly converted to 25-hydroxyvitamin D3 [25(OH)D3] by 25-hydroxylase in the liver, but it becomes metabolically active when converted to 1,25-dihydroxyvitamin D3 [1,25(OH)2D3] by 1-α-hydroxylase in the kidney (7). Both a deficiency and an excess of vitamin D can be harmful. Non-dietary and dietary intoxications have already been occasionally reported in cats due to the accidental ingestion of vitamin D3-containing rodenticides, along with the over-supplementation of vitamin D2 and/or D3 in cat foods (8–10). The aim of this case series is to describe the clinical presentation, diagnostic findings, and outcome of an acute intoxication that occurred in five young cats fed a commercial kitten food characterized by excessive vitamin D3 content. More precisely, the cat food included the highest amount of vitamin D3 that, to the authors knowledge, has ever been reported to be fed to domestic cats. Moreover, the fact that it was eaten as the sole food by two of these cats, despite being declared as complementary food (which means that it is intended to only be a part of the diet, as it does not meet all of the requirements of essential nutrients), poses concerns about the correct use and feeding recommendations of complementary pet foods (11).

## Case series description

Five domestic shorthair young cats (one intact male, two castrated males, and two spayed females) ranging from 2 to 17 months and a body weight varying from 850 to 4,000 g, living separately (except for two of such cats within the same household), were all presented in two veterinary referrals in Italy within a 1-month period showing similar clinical signs. The most common gastrointestinal signs described were anorexia (described for 5/5), vomiting (4/5), followed by weakness (2/5, case 2 and 4), and increased respiratory effort and cough; the latter was only reported for a 5-month-old cat (case 2) and was the sole symptom. Moreover, this cat presented increased lung sounds on expiration, while the physical examination was unremarkable for the other cats.

At the first presentation, a diagnostic workup including a complete blood count, serum biochemistry, and venous blood gas analysis was performed for all of the cats except for the younger one (case 1), which, due to his small size, was tested only for a venous blood gas analysis and serum urea. The selected blood parameters and urine specific gravity—revealed at first presentation—are summarized (Table 1). All of the cats presented a severe increase of ionized calcium (iCa) and total serum calcium (Ca), except for case 1 where the Ca value was not available. However, cases 1 and 4 presented hyperkalemia [*K* = 4.71 and 4.60 mmol/L, respectively (reference values 2.90–4.50 and 3.50–4.50 mmol/L, respectively)], while other cats revealed a normal blood potassium level. Hyperphosphatemia was detected in case 2 and case 4 [P = 7.40 and 7.60 mg/dl, respectively (reference values 2.60–6.20 and 2.50–6.20 mg/dl, respectively)], although in all of the cats, calcium × phosphate (Ca × P) product exceeded 60 mg/dl (a value frequently quoted by some authors as being the safe threshold against the metastatic mineralization of soft tissues) (10, 12–14). Nevertheless, higher values of Ca × P should be considered physiologically normal for growing animals, since young cats (cases 2 and 3) could have physiologically elevated baseline serum calcium and phosphorus levels due to enhanced intestinal mineral absorption and decreased renal excretion, presumably to facilitate bone mineralization (15, 16). Case 4 and case 5 presented notably increased levels of both serum creatinine [5.04 and 2.31 mg/dl, respectively (reference value 0.8–1.80 mg/dl)] and urea [185.30 and 69.99 mg/dl, respectively (reference value 15–60 mg/dl)], while case 2 showed increased urea [103 mg/dl (reference value 32–64 mg/dl)] with a normal creatinine level. A free-catch urine sample was only obtained from cases 4 and 5 to evaluate the urine-specific gravity measures that were inappropriately low for the degree of dehydration and azotemia (1.014 and 1.030, respectively) and they were consistent with impaired concentrating ability.

**Table 1:** Results of selected blood parameters and urine specific gravity at first presentation and follow-up for cases 1, 3, 4, and 5 (the days since the first presentation are indicated).

	Case 1*		**Case 2**	**Case 3**		**Case 4**						**Case 5**					**Reference value**
	**(m, 2 m)**		**(mC, 5 m)**	**(mC, 7 m)**		**(mC, 10 m)**						**(fS, 17 m)**					
**Days**	**0**	**10**	**0**	**0**	**3**	**0**	**4**	**10**	**12**	**16**	**32**	**0**	**2**	**7**	**10**	**33**	
Creatinine (mg/dl)	NA	NA	1.58^†^	1.20^†^	NA	5.04^‡^	8.66^‡^	7.13^‡^	5.29^‡^	4.98^‡^	4.65^‡^	2.31^‡^	2.02^‡^	3.47^‡^	2.99^‡^	2.26^‡^	^†^0.6–1.85;
																	^‡^0.8–1.80
Urea (mg/dl)	49^†^	NA	103^†^	60^†^	NA	185.3^‡^	NA	NA	172.6^‡^	NA	142.3^‡^	69.99^‡^	82.45^‡^	104.4^‡^	NA	100.4^‡^	^†^32–64;
																	^‡^15–60
K (mmol/l)	4.71^†^	NA	NA	3.53^†^	NA	4.60^‡^	NA	NA	NA	NA	NA	4.40^‡^	NA	NA	NA	NA	^†^2.90–4.50;
																	^‡^3.50–4.50
iCa (mmol/l)	2.37^†^	1.42^†^	1.89^†^	1.95†	1.72†	1.87^‡^	NA	NA	NA	NA	NA	2.09^‡^	NA	1.52^‡^	NA	NA	^†^1.20–1.32;
																	^‡^1.24–1.41
Ca (mg/dl)	NA	NA	16.8^†^	16.8^†^	NA	17.03^‡^	14.08^‡^	12.51^‡^	10.73^‡^	12.07^‡^	12.77^‡^	16.68^‡^	18.48^‡^	16.47^‡^	14.43^‡^	11.74^‡^	^†^7.30–11.20;
																	^‡^6.0–10.5
P (mg/dl)	NA	NA	7.40^†^	4.90^†^	NA	7.6^‡^	NA	NA	NA	NA	NA	4.50^‡^	4.1^‡^	4.80^‡^	NA	4.90^‡^	^†^2.60–6.20;
																	^‡^2.5–6.2
Ca × P (mg^2^/dl^2^)	NA	NA	124	82.32	NA	129.4	NA	NA	NA	NA	NA	75.06	75.77	79.05	NA	57.52	<60
Urine specific gravity	NA	NA	NA	NA	NA	1.014	NA	NA	NA	NA	NA	1.030	NA	NA	NA	NA	>1.040

m, male; mC, castrated male; fS, spayed female; xm, cat's age expressed in months; Ca, total calcium; Ca × P, calcium × phosphate product; iCa, ionized calcium; K, potassium; NA, not available; P, phosphate. The laboratory reference intervals are indicated with different symbols (^†^ and ‡); * in case 1, the treatment only consisted of pet food withdrawal.

**Figure 1:**
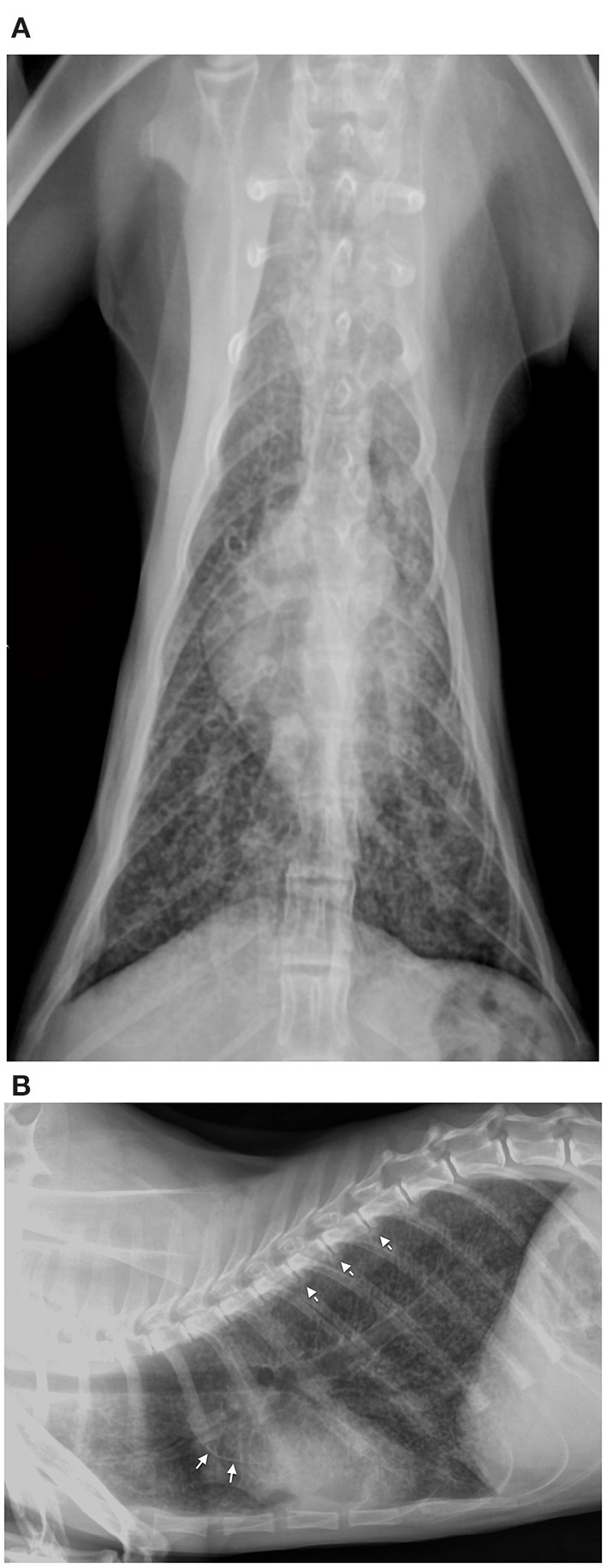
Ventrodorsal **(A)** and right lateral view **(B)** thoracic radiographs of hypercalcemic case 2 revealed increased lung volume and a diffuse bronchointerstitial pattern with structured peri-bronchial mineralization. Furthermore, multiple areas of mineralization were visible along the aortic arch (arrow) and the ventral surface of the thoracic vertebral bodies (dotted arrow).

Diagnostic imaging was performed in cases 2, 4, and 5; the radiograph study on case 2 (Figures 1A,B) showed increased lung volume and a diffuse bronchointerstitial pattern with structured peri-bronchial and interstitial mineralization. Moreover, multiple areas of bone radiopacity were visible at the aortic arch, along the ventral surface of the thoracic vertebral bodies, within the gastric wall. The thoracic radiographs of case 4 (see Supplementary Figures 1A,B) and case 5 showed a mild and diffuse bronchial pattern that is consistent with inflammation or an allergic disease associated with the increased radiopacity of the wall of main bronchi. The abdominal ultrasonography performed in cat 4 revealed reduced renal corticomedullary distinction and mild pyelectasis while cat 5 showed diffuse and focal small bowel inflammatory enteropathy and diffuse splenopathy; the latter is consistent with a hyperplastic or reactive condition.

A complete nutritional history was recorded for all of the cats. It was noted that the same canned complementary kitten food was fed to all of the cats as part of their diet (cases 1, 2, and 3) or consumed as the primary diet (cases 4 and 5); case 1 received one tin (70 g) per day of this kitten pet food (KPF), cases 2 and 3 received two tins per day of KPF, while cases 4 and 5 were fed exclusively with KPF (three and four tins per day, respectively). Excessive vitamin D intake from this KPF was suspected, and therefore, it was subsequently investigated. The labeled composition of KPF, marketed as complementary food for growing kittens without additives, consisted of skipjack tuna (*Katsuwonus pelamis*) 49%, eggs 20%, water 18%, skipjack tuna-liver 5%, skipjack tuna-oil 5%, sunflower oil 2%, and rice 1%. Analytical constituents declared crude protein 16%, crude fat 5%, crude fiber 1%, crude ash 2%, moisture 76%, and metabolizable energy content 985 kcal/kg.

Based on the suspicion of vitamin D toxicity, two batches of KPF were sent to a laboratory for the quantification of the vitamin D3 content by high-performance liquid chromatography (HPLC) as well as moisture (AOAC 934.01), crude ash (AOAC 942.05), calcium (ISO 6869:2000), and phosphorus (AOAC 965.17) content. The stated label and measured amounts are presented in Table 2. The analytical values of moisture and crude ash were discordant from the labeled composition, but the deviations were within the tolerances permitted by EU legislation (11). Kitten pet food resulted in markedly elevated vitamin D3 content (27,300 IU/100 g on an as fed basis). Therefore, for each cat, a comparison was made between the vitamin D3 intake from KPF, the safe upper limit for vitamin D3 recommended by NRC (17), and the vitamin D nutritional maximum recommended by FEDIAF (18). The results are shown in Table 3. The daily energy requirement was calculated for each cat according to NRC equations, and then energy and vitamin D3 intake from KPF were assessed (Table 3): energy intake derived from KPF was different in cats, ranging from 38% (case 1) to 109% (case 4) of the daily energy requirement, and consequently, the vitamin D3 intake was different according to the amount of KPF fed, with a mean intake per day of 45,176 IU.

**Table 2:** Declared vs. measured moisture, crude ash, minerals, and vitamin D3 content of kitten pet food (batch A and B). The data are expressed as %, or IU/100 g, of OS.

	**Stated label amount**	**KPF batch A**	**KPF batch B**	Mean ±SD^*^
Moisture (%)	76.0	79.33	78.92	79.13 ± 0.28
Crude ash (%)	2.0	1.09	1.17	1.13 ± 0.06
Calcium (%)	–	0.02	0.02	0.02 ± 0
Phosphorus (%)	–	0.14	0.14	0.14 ± 0
Vitamin D3 (IU)	–	35,600	19,000	27,300 ± 11,738

*KPF, kitten pet food; OS, original substance (as fed basis); IU, international unit.*

* *Data are expressed as the mean value ± SD of the two batches*.

**Table 3:** Daily energy and vitamin D3 intake from kitten pet food, safe upper limit for vitamin D3, nutritional maximum for vitamin D, and the percentage difference for each cat.

	**Case 1**	**Case 2**	**Case 3**	**Case 4**	**Case 5**	Mean ±SD^*^
Body weight (kg)	0.85	2.35	3.20	4.00	3.10	2.70 ± 1.06
Daily energy requirements (kcal ME/day)^a^	180	280	292	253	213	243 ± 41.3
Daily energy intake from KPF (% of daily energy requirements)^b^	38	49	47	109	97	68 ± 28.9
vitamin D3 intake (IU/cat) from KPF^c^	18,823	37,647	37,647	75,293	56,470	45,176 ± 19,196
SUL for vitamin D3 (IU/cat; NRC, 2006)^d^	1,218	3,719	4,781	1,924	1,622	2,653 ± 1,365
Δ% between vitamin D3 intake (KPF) and SUL	1,445	912	687	3,813	3,382	–
Nutritional maximum for vitamin D (IU/cat; FEDIAF, 2020)^e^	1,355	2,092	2,187	1,899	1,601	1,827 ± 310
Δ% between vitamin D3 intake (KPF) and the FEDIAF nutritional maximum for vitamin D	1,289	1,699	1,621	3,866	3,428	–

* *Data are expressed as the mean value ± SD; KPF, kitten pet food.*

a *Daily metabolizable energy requirement calculated by the NRC equations proposed for growth kittens after weaning (cases 1, 2, and 3) and adult cats at maintenance (cases 4 and 5).*

b *KPF energy density: 98 kcal/100 g as fed.*

c *Based on measured KPF vitamin D3 content IU/100 g = 27,300.*

d *SUL = safe upper limit (IU vitamin D3/cat).*

e *Established on the nutritional maximum for vitamin D (D2 + D3) recommended by FEDIAF = 7,500 IU/1,000 kcal ME*.

Based on the previously described calculations of the daily energy requirement, the NRC safe upper limit established for vitamin D3 (IU/cat) and the FEDIAF nutritional maximum established for vitamin D (IU/cat) were calculated for each animal: the vitamin D3 mean intake resulted in more than 40 times above the safe upper limit and more than 43 times above the nutritional maximum intake of vitamin D. Case 4 ingested the highest daily vitamin D3 amount (75,293 IU/day), followed by case 5 (56,470 IU/day).

All of the cats were treated with KPF withdrawal and the medical management of hypercalcemia, except for case 1 that was treated only with KPF discontinuation. Cats were hospitalized for 1 (cases 1 and 2), 3 (case 3), 5, and 14 days (cases 4 and 5, respectively) and have been managed with intravenous fluid therapy with a ringer lactate solution at a rate of 2–4 ml/kg/h IV q24h, administration of sucralfate (30 mg/kg PO q24h, Antepsin; Baldacci Laboratori), prednisolone (1 mg/kg q24h IV, VetSolone; Bayer), and omeprazole (0.7 mg/kg IV q24h, Antra; AstraZeneca). After 2 days, iCa remained elevated in cases 4 and 5. Therefore, these cats were also treated with furosemide (2 mg/kg q12h IV, Diuren Teknofarma) and switched to fluid therapy with isotonic saline at a rate of 1 to 2 ml/kg/h IV q24h (0.9% sodium chloride; B Braun). After the introductory treatment, iCa in cases 1, 3, and 5 and total serum calcium and renal function parameters in azotemic cats (cases 4 and 5) have been measured and follow-up results are presented in Table 1: iCa was tested again only once more in cases 1, 3, and 5, and all of the values were above the upper limit even after 10 days of KPF withdrawal. A follow-up of case 2 was not possible due to the owner's decision. About 1 month after initiating medical treatment, cases 4 and 5 remained markedly azotemic (creatinine 4.65 and 2.26 mg/dl; urea 142.30 and 100.40 mg/dl, respectively); although the total serum calcium levels decreased gradually, it remained above the upper limit (12.77 and 11.74 mg/dl, respectively; upper limit 10.5 mg/dl).

One month after recovery, radiographic findings in case 2 showed the improvement of pulmonary mineralization, while the vertebral exostosis worsened.

Following KPF discontinuation, a nutritional assessment was performed for all of the cats: due to his young age, case 1 exclusively consumed dry kitten food, whereas the other cats were fed calcium- and vitamin D-restricted diets for at least 30 days after diagnosis. Based on the owners' preferences, cases 2 and 3 received complementary wet cat food, while cases 4 and 5 were initially fed a chicken and rice-based homemade diet and were later switched to renal pet food.

As a result of medical therapy and KPF withdrawal, all of the clinical signs resolved rapidly in cases 1, 2, and 3, while a diminished appetite in addition to polyuria and polydipsia persisted in cases 4 and 5, likely secondary to chronic kidney disease. Serial follow-ups were performed in cases 4 and 5 in subsequent years (data not shown), and, based on the concentration of serum creatinine, they were both classified as IRIS stage 2 (19).

## Discussion

In this case series, acute dietary vitamin D3 toxicosis has been described in five young cats. Although not common, dietary vitamin D3 toxicity has even been reported in domestic cats and captive felids (tigers and lynxes) recently, thereby resulting in renal failure and widespread mineralization in various organs (4, 5, 12, 13, 20, 21).

Besides felids, induced—or naturally occurring—vitamin D intoxications have been reported in a variety of different species, including poultry, rabbits, dogs, horses, and rats (22–31). Hypercalcemia, hyperphosphatemia, and ectopic mineralization are consistent findings in mammalians, while in poultry, mainly renal tubular mineralization is described (23); however, in young broiler chicks, a vitamin D3 intake higher than 50,000 IU/kg showed no apparent negative effects (24). Considering mammalians, calcific deposits in the aorta and kidney arose in rabbits by giving 10,000 IU vitamin D3/kg of diet per day, while a total dose of 600,000 IU of vitamin D caused young rabbits to die within 6 weeks (22, 26). Similarly, a daily administration of 100,000 UI of vitamin D3 in dogs was fatal within 60 days (25). In horses, the regulation of calcium homeostasis is not as dependent on vitamin D as in other species. Therefore, they have low basal vitamin D metabolite levels that increase much lower compared to humans and rodents when vitamin D intoxication occurs (27); persistent hyperphosphatemia rather than hypercalcemia was recognized in horses fed more than 1,000,000 IU of vitamin D3/kg for about 30 days (28), and this finding was also described more recently (27). In rats, daily injections of 2.5 or 5 UI of 1,25(OH)2D3 induced hypercalcemia and the consequent ectopic mineralization was enhanced by high-calcium diets (29). Moreover, in this species, it was demonstrated that dietary vitamin D3 excess (10,000 IU/kg of diet) enhances circulating 25(OH)D3, rather than 1,25(OH)2D3, probably because the latter is strictly modulated by the renal enzyme 1-α-hydroxylase even when vitamin D ingestion is much higher than the optimal requirement (30–32). Similar to what has been described in rats, an increased concentration of 25(OH)D3 in response to the dietary intake of vitamin D was observed also in kittens and, therefore, a large reserve activity of enzymatic hydroxylase in the liver could be one of the tolerance mechanisms that protect this species against excessive vitamin D3 intake (33).

On the contrary, low dietary calcium leads to insufficient intestinal absorption of calcium and low plasma calcium levels stimulate the release of the parathyroid hormone (PTH), which causes bone resorption of calcium and phosphorus, while the kidney activates 1,25(OH)2D3, which in turn increases the absorption of calcium and phosphorus (34, 35). However, low dietary calcium content in the presence of constant vitamin D intake may cause depletion of 25(OH)D3, as a consequence of the high activity of the renal enzyme 1-α-hydroxylase, which converts 25(OH)D3 into 1,25(OH)2D3 (36).

In the present case series, the calcium content of KPF was largely lower than the minimum level recommended by FEDIAF, and low calcium intake may have been beneficial in terms of a potentially protective role against more severe effects derived from vitamin D3 toxicity, especially in the case of the two cats fed exclusively with KPF, even if impaired renal function was confirmed after serial measurements.

The vitamin D3 amount in cat food detected in previous reports ranged from 46,900 to 67,300 IU/kg of food (4, 14), while in this case series, an extremely high vitamin D3 content (273,000 IU/kg of food) was detected, four-fold higher than the levels reported in the abovementioned studies (Table 2). In a previous study (37), an experimental diet high in vitamin D3 (33,840 IU/kg) was fed to young cats and queens for 18 months to determine the effects on reproduction, growth, and renal health; at the end of the trial, the serum calcium and renal parameters were unaffected. The authors speculated that other factors may contribute to the severity of vitamin D3 toxicity, including dietary calcium, phosphorus, and magnesium intake (37).

Through evolutionary adaptation to the consumption of prey rich in vitamin D, including fish, fat, liver, offal, and blood, carnivorous animals lost their ability to synthesize vitamin D from the skin (resulting in inefficient conversion from 7-dehydrocholesterol to vitamin D3) and, therefore, they solely rely on dietary intake (38). Compared to dogs, cats cannot convert vitamin D2 as efficiently as they can convert vitamin D3 (4) and this may be considered another protective mechanism against vitamin D toxicity, probably due to the high activity of the C3-epimerization pathway, a recently discovered alternative metabolism pattern of vitamin D that leads to the production of 3-epi-1,25(OH)2D3, as an epimer of 1,25(OH)2D3. The 3-epi-1,25(OH)2D3 shows reduced biological activity compared to 1,25(OH)2D3; in fact, it seems that 3-epi-1,25(OH)2D3 is able to suppress PTH secretion equally vs. its counterpart without inducing the other usual calcemic effects (39). In addition, other pathways for vitamin D, initiated by enzymes of the cytochrome P450-family and regulated by calciotropic hormones, have recently been discovered in producing inactive, or less physiologically active, metabolites (40, 41). Among these, the major product of CYP11A1, 20(OH)D3, is usually present at low endogenous levels and functioning in specific target tissues, such as either human serum, epidermis, and placenta or murine and porcine adrenal glands, and has shown *in vitro* and *in vivo* non-calcemic and non-toxic effects, including at extremely high doses, thereby suggesting a potential therapeutic use for the treatment of hyperproliferative and inflammatory disorders in humans (42–44).

In the present case series, serum PTH, PTH-related protein, 25(OH)D3, and 1,25(OH)2D3 were not measured, which constitutes a limitation of this study since the decrease of PTH, increase of 25(OH)D3 and 1,25(OH)2D3, in addition to hypercalcemia and hyperphosphatemia, are confirmatory findings of vitamin D toxicity (12). However, in this study, all of the cats presented elevated Ca × P product, in addition to the radiographic evidence of the mineralization of soft tissues in case 2. A Ca × P product of >60 mg/dl is reported by veterinary textbooks and guidelines for hypercalcemia management as the level considered as risky for soft tissue mineralization (10, 13). This value is not a specific threshold established for cats, but it has been simply extrapolated from human medicine (45), in which it is considered as an indicator for ectopic calcifications in end-stage chronic renal disease patients (46–48), although other authors have not supported a real correlation between metastatic calcification and the level of the Ca × P product, as soft tissue mineralization seems to be a complex process in which other factors, e.g. azotemia and a loss of endogenous calcification inhibitors, seem to be involved (49, 50).

Kitten pet food was fed as the only food to cases 4 and 5, while it was declared as a complementary food that, by its definition, should be fed only in combination with other pet foods to fulfill all of a cat's nutritional requirements (11). The vitamin D3 mean daily intake of cases 4 and 5 was more than two-fold higher than the mean intake obtained from cases 1, 2, and 3 (65,882 vs. 31,372 IU, respectively). Moreover, apart from excessive vitamin D3 content, cases 4 and 5 were exposed to other nutritional imbalances, including inadequate calcium intake and an inverted calcium:phosphorus ratio (Ca:P). On a dry matter basis, the minimum levels recommended by FEDIAF for calcium and the Ca:P ratio in complete cat foods are 0.53% and 1 (18), respectively, while calcium and Ca:P ratio values in KPF were 0.09% and 0.14, respectively. Insufficient dietary calcium content or an inadequate Ca:P ratio can be associated with secondary nutritional hyperparathyroidism, loss of bone, and osteopenia in long-term periods (51).

KPF contained vitamin D-rich ingredients, such as fish liver and fish oil. The published data show that the highest values of vitamin D in food are found in fish and especially in fish liver, ranging from <80 to 19,080 IU/kg and up to 48,000 IU/kg, respectively (52), depending on the fish species and farming systems (the differences in climate, food supply, and nutritional condition, as regards the vitamin D3 content of plankton, provide reasons for these variations) (53, 54); in particular, skipjack tuna (*K. pelamis*), the main ingredient of KPF, typically contains 7,500 IU vitamin D3/kg (52). If vitamin D is added to a pet food as an additive, the added amount, rather than the total amount, has to be declared within the additives list [according to Regulation (EC) No. 1831/2003] (55) and it should be lower than 30,000 IU/kg of food on a dry matter basis (10). In this case, vitamin D3 in KPF reached 1,311,770 IU/kg on a dry matter basis, exceeding 44-fold the recommended maximum nutritional level by FEDIAF. Despite the presence of fish liver and fish oil in KPF, such a high concentration of vitamin D is not consistent with the ingredients list. Moreover, KPF was labeled as additive-free and, therefore, it should be presumed that no vitamin D was added. The amounts of vitamin D found in KPF were too high even if cod liver oil, instead of fish oil, had been accidentally used (however, the authors do not have any evidence of the presence of cod liver oil in KPF).

According to the producer's opinion, a not well-defined vitamin D3 contamination of raw materials was the most likely cause of its high levels. As a result, the producer carried out a voluntary market recall of all batches of KPF.

Most of the cases showed the typical clinical presentation (anorexia, apathy, polyuria, polydipsia, and gastrointestinal signs) associated with acute vitamin D3 toxicosis in companion animals (12); at first presentation, instead, case 2 exhibited, as a main clinical sign, a moderate respiratory distress (increased respiratory effort and cough), in addition to anorexia. Respiratory distress could be due to a pulmonary calcification that is able to induce lung injury by disrupting the normal alveolar-capillary wall, as already reported in humans (14).

From a histological perspective, acute vitamin D toxicity could also be related to liver and kidney damage resulting from 1,25(OH)2D3-induced distress on hepatocytes and the activation of defense mechanisms on kidney cells, as observed recently in hamsters being tested with a high dose of 1,25(OH)2D3 (56).

A moderate increase in serum phosphorus (up to 10 mg/dl) with a rapid and more severe increase in serum calcium (up to 20 mg/dl) usually occurs 12–72 h following consumption; serum urea and creatinine levels also tend to increase shortly after excessive vitamin D3 ingestion (57). In this case series, at first evaluation, urea was impaired except for cases 1 and 3, while total serum calcium (and ionized calcium) increased promptly in addition to the Ca × P product.

Rehydration is an essential component in the treatment of feline severe hypercalcemia (when Ca > 1.6 mmol/dl) (58), as it has a double effect: fluid therapy (4–6 ml/kg/h) decreases serum calcium concentrations by correcting the dehydration and it allows calciuresis to occur by limiting renal reuptake (12). In the presence of correct hydration, the diuretic furosemide (1–2 mg/kg q8–12h) helps to inhibit renal calcium reuptake, while prednisolone (1–3 mg/kg q8–12h) can be administered to exacerbate renal calcium excretion (12). Although not given to these cats, bisphosphonates are considered the mainstay of treatment for hypercalcemia because they inhibit calcium release from bone by the induction of osteoclast apoptosis (59).

Due to its fat solubility, vitamin D is accumulated in fat tissue and may further be metabolized slowly for several weeks to months, while soft tissue mineralization is minimally reversible and can lead to organ structural damage (56). In this case series, serum calcium measurements were not performed in all cats, but cases 4 and 5 were still showing high serum calcium levels after more than 1 month following the withdrawal of KPF, while a previous report described return to normocalcemia within 19 days (9). These two cats developed kidney failure due to vitamin D3 toxicosis and may, therefore, suffer from the impaired renal excretion of vitamin D metabolites.

## Conclusion

To the authors' knowledge, the present study describes the highest amount of dietary vitamin D3 intake that has ever been reported in cats. Vitamin D supplementation in pet food is not risk-free, and it should be carefully monitored by pet food manufacturers, while clinicians should be aware that dietary vitamin D toxicity, although uncommon, can arise.

## Data Availability Statement

The original contributions presented in the study are included in the article/Supplementary Material, further inquiries can be directed to the corresponding author/s.

## Ethics Statement

Ethical review and approval was not required for the animal study because this case series describes medical treatments and diagnostics performed on privately owned animals. Written informed consent was obtained from the owners for the participation of their animals in this study.

## Author Contributions

GG, MP, and LZ directly managed diagnosis and treatment of the cases. CP and CV performed analyses on pet food. GB and CD contributed to data interpretation. SP reviewed and interpreted the imaging. CV wrote the report with assistance and feedback from SP, CP, and MS. GB, MP, CP, and MS critically revised the report. CD contributed to editing the report. GG and LZ reviewed the final submission. All authors contributed to the article and approved the submitted version.

## Conflict of Interest

The authors declare that the research was conducted in the absence of any commercial or financial relationships that could be construed as a potential conflict of interest.

## Publisher's Note

All claims expressed in this article are solely those of the authors and do not necessarily represent those of their affiliated organizations, or those of the publisher, the editors and the reviewers. Any product that may be evaluated in this article, or claim that may be made by its manufacturer, is not guaranteed or endorsed by the publisher.
