# Supplementary material for: Case Report: A Case Series Linked to Vitamin D Excess in Pet Food: Cholecalciferol (Vitamin D3) Toxicity Observed in Five Cats
Source: Front Vet Sci. 2021 Aug 18;8:707741. doi: 10.3389/fvets.2021.707741 (PMC8416511; doi:10.3389/fvets.2021.707741)
Supplement: Supplementary file 1 [file Data_Sheet_1.zip › Supplementary_Material.docx]

Supplementary Material

# Supplementary Figures

**Supplementary Figure 1 (A) and (B).** Ventro-dorsal (A) and right lateral view (B) thoracic radiographs of a hypercalcemic cat (case 4), revealed mild and diffuse bronchial pattern, consistent with inflammation or allergic disease. Mild increased radiopacity of main caudal right bronchus is also visible.
