# Supplementary figures and images for: Case Report: A Case Series Linked to Vitamin D Excess in Pet Food: Cholecalciferol (Vitamin D3) Toxicity Observed in Five Cats
Source: Front Vet Sci. 2021 Aug 18;8:707741. doi: 10.3389/fvets.2021.707741 (PMC8416511; doi:10.3389/fvets.2021.707741)

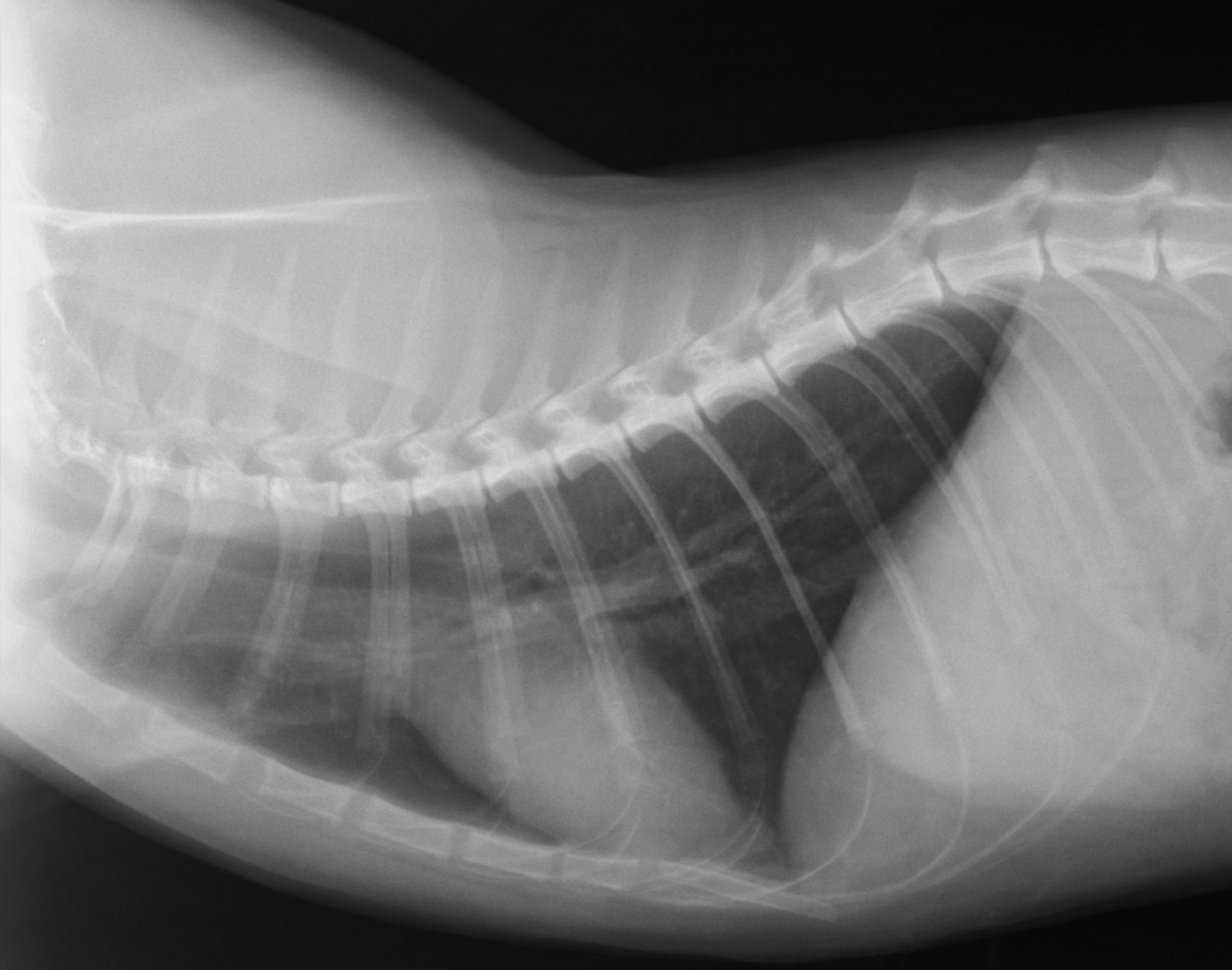

Supplement: Supplementary file 1 [file Data_Sheet_1.zip › Supplementary Figure 1A.tif]

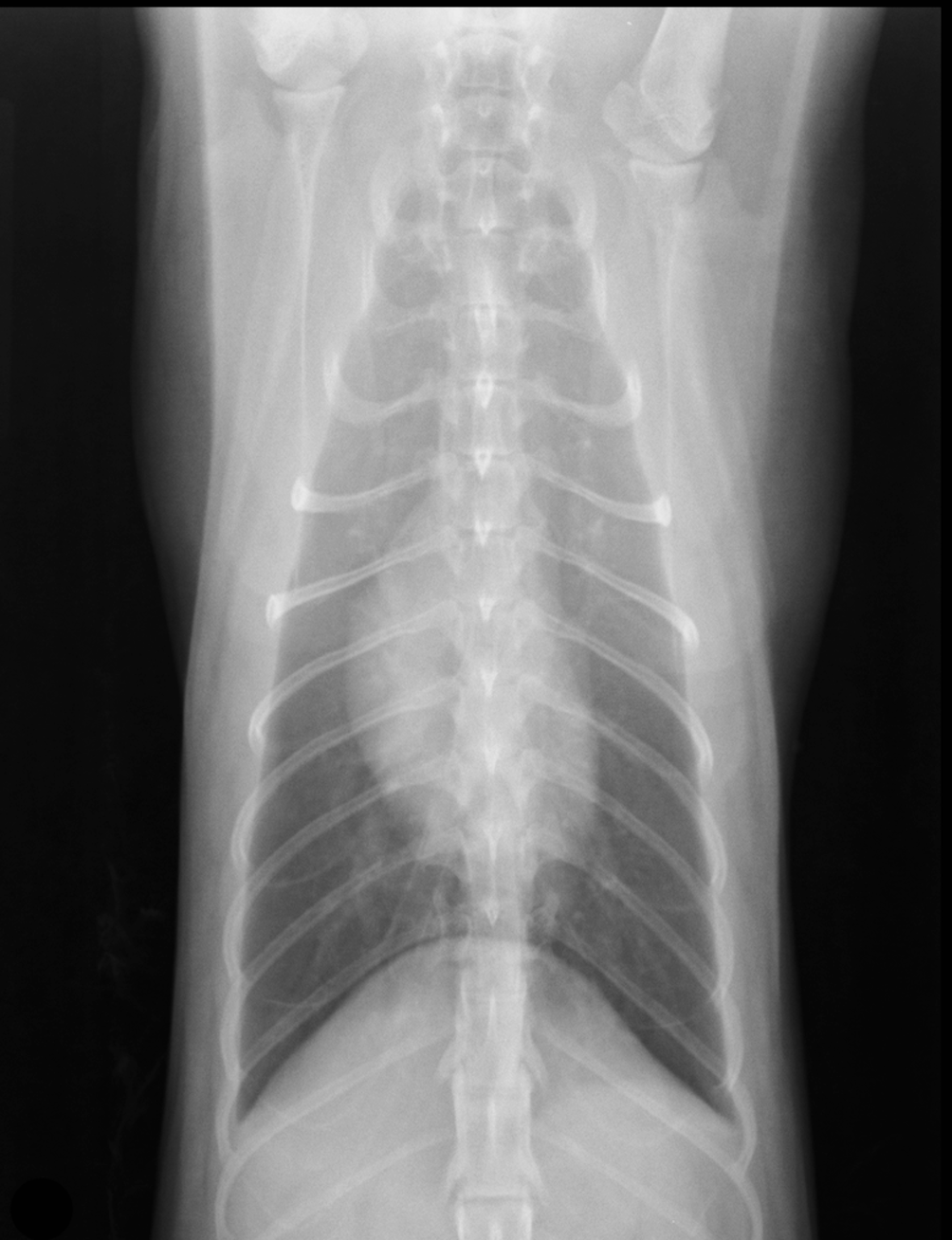

Supplement: Supplementary file 1 [file Data_Sheet_1.zip › Supplementary Figure 1B.tif]
